# Accurate assembly of thiophene-bridged titanium-oxo clusters with photocatalytic amine oxidation activity†

**DOI:** 10.1039/d4ra00117f

**Published:** 2024-03-06

**Authors:** Haoran Nai, Jinle Hou, Jinyu Li, Xiaoxi Ma, Yujia Yang, Konggang Qu, Xianqiang Huang, Lianzhi Li

**Affiliations:** a Shandong Provincial Key Laboratory of Chemical Energy Storage and Novel Cell Technology, School of Chemistry and Chemical Engineering, Liaocheng University Liaocheng 252000 People's Republic of China houjinle@lcu.edu.cn hxq@lcu.edu.cn lilianzhi1963@163.com

## Abstract

Designing and synthesizing well-defined crystalline catalysts for the photocatalytic oxidative coupling of amines to imines remains a great challenge. In this work, a crystalline dumbbell-shaped titanium oxo cluster, [Ti_10_O_6_(Thdc)(Dmg)_2_(^i^PrO)_22_] (Ti10, Thdc = 2,5-thiophenedicarboxylic acid, Dmg = dimethylglyoxime, ^i^PrOH = isopropanol), was constructed through a facile one-pot solvothermal strategy and treated as a catalyst for the photocatalytic oxidative coupling of amines. In this structure, Thdc serves as the horizontal bar, while the {Ti_5_Dmg} layers on each side act as the weight plates. The molecular structure, light absorption, and photoelectrochemical properties of Ti10 were systematically investigated. Remarkably, the inclusion of the Thdc ligand, with the assistance of the Dmg ligand, broadens the light absorption spectrum of Ti10, extending it into the visible range. Furthermore, the effective enhancement of charge transfer within the Ti10 was achieved with the successful incorporation of the Thdc ligand, as opposed to PTC-211, where terephthalic acid replaces the Thdc ligand, while maintaining consistency in other aspects of Ti10. Building on this foundation, Ti10 was employed as a heterogeneous molecular photocatalyst for the catalytic oxidative coupling reaction of benzylamine (BA), demonstrating very high conversion activity and selectivity. Our study illustrates that the inclusion of ligands derived from Thdc enhances the efficiency of charge transfer in functionalized photocatalysts, significantly influencing the performance of photocatalytic organic conversion.

## Abstract 2

We synthesized a crystalline dumbbell-shaped Ti10, which effectively enhances charge transfer. As a result, it exhibits remarkably high conversion activity and selectivity in the photocatalytic oxidative coupling reaction of benzylamine.

## Graphical abstract

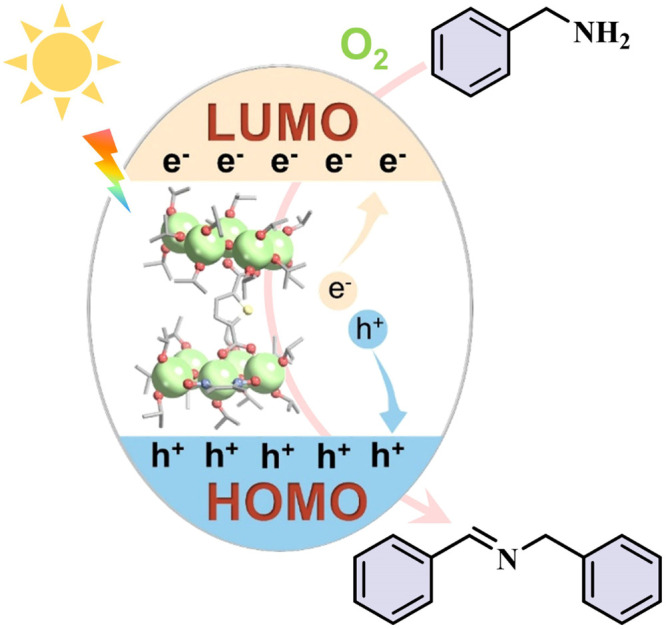

## Introduction

Photocatalytic organic conversion stands out as a prospective method for achieving direct chemical bond functionalization in ambient conditions. This hopeful strategy has the potential to simultaneously address pressing concerns about the energy crisis and environmental issues, driving the sustainable and high-quality advancement of the chemical industry.^1–4^ Within these photocatalytic organic conversions, the oxidative coupling of amines under molecular oxygen conditions holds great significance in generating high-value imines, serving as crucial organic intermediates for the synthesis of biologically active compounds and fine chemicals.^5–7^ Conventional approaches to imine preparation often require harsh reaction conditions, involving high temperatures and pressures, leading to inevitable environmental pollution.^8,9^ The photocatalytic oxidative coupling of amines, conversely, emerges as a compelling alternative owing to its inherent merits: straightforward operation, gentle reaction conditions, cost-effectiveness, and environmental friendliness.^10–12^ To date, a range of photocatalysts, such as metal sulfides, metal oxides, and TiO_2_-organic hybrids, have been reported for the oxidative coupling of amines to imines, achieving significant advancements.^13–15^ However, most of these catalysts always lack clear structural information and exhibit complex interfacial information, posing substantial challenges in comprehending the connection between structure and functionality. Therefore, the development of well-defined crystalline photocatalysts is an urgent need.

Titanium oxo clusters (TOCs), as molecular models of TiO_2_ nanoparticles, have rapidly developed. TOCs not only exhibit similar catalytic activity to TiO_2_, but also readily grow into single crystals, facilitating structure identification. Furthermore, their well-defined structural information provides a crucial foundation for establishing an efficient structure–property relationship at the molecular level.^16–20^ Specifically, as an aggregate containing multiple metal ions, TOCs can generate multiple active metal sites by judiciously manipulating the coordination environment of metal ions.^21^ This, in turn, offers increased possibilities for enhancing catalytic activity and facilitating the application of photocatalytic reactions. Recently, several research groups have achieved significant advancements in the photocatalytic performance of TOCs, encompassing hydrogen production, dye degradation, and CO_2_ reduction reaction (CO_2_RR).^22–27^ However, to the best of our knowledge, there is a scarcity of reported TOCs employed in the photocatalytic oxidative coupling of amines to imines.^28^ For instance, Liu *et al.* explored the photocatalytic activity of TOCs in the oxidative coupling of amines, utilizing ferrocene-functionalized TOCs for the first time.^29^ Therefore, more research efforts should be directed towards synthesizing novel TOCs for the photocatalytic oxidative coupling of amines. Nevertheless, numerous challenges persist in the utilization of conventional TOCs in photocatalytic applications. The primary drawback lies in their typically wide band gaps, rendering them responsive solely to ultraviolet light.^30–32^ To address this, researchers focus on regulating the band gap through ligand modification, thereby tailoring the light absorption range.^33,34^ Therefore, the selection of suitable functionalized organic ligands becomes crucial for designing novel TOCs with broadened light absorption.

The derivatives of thiophene (Th), which serve as sulfur-containing heterocyclic moieties, exhibit electron-rich characteristics, enabling their use as electron donors. Their outstanding electronic properties have led to widespread use in electronic and photoelectronic devices.^35–37^ For instance, polythiophene derivatives have been used in organic solar cells.^38,39^ However, only a limited number of TOCs containing Th-derived functional ligands have been documented to date.

Based on the aforementioned factors, we designed and prepared a dumbbell-shaped TOC, [Ti_10_O_6_(Thdc)(Dmg)_2_(^i^PrO)_22_] (Ti10, Thdc = 2,5-thiophenedicarboxylic acid, Dmg = dimethylglyoxime, ^i^PrOH = isopropanol), employing solvent thermal methods. In this structure, Thdc serves as the horizontal bar, while the {Ti_5_Dmg} layers on each side act as the weight plates. Remarkably, the coordination of Thdc and Dmg ligands extends the light absorption range of Ti10 into the visible light region. Furthermore, the inclusion of the Thdc ligand notably enhances the photocurrent response of Ti10 in comparison to PTC-211. In PTC-211, terephthalic acid replaces the Thdc ligand, while other aspects remain consistent with Ti10.

Given these advantages, we proceeded with additional research on the photocatalytic oxidative coupling reaction of benzylamine (BA) using Ti10. Notably, Ti10 demonstrated exceptional light-driven photocatalytic efficiency, achieving a 99% yield and selectivity under ambient pressure. We further propose a plausible photocatalytic mechanism for this reaction.

## Results and discussion

### Syntheses and structure analyses of Ti10

**Scheme 1:**
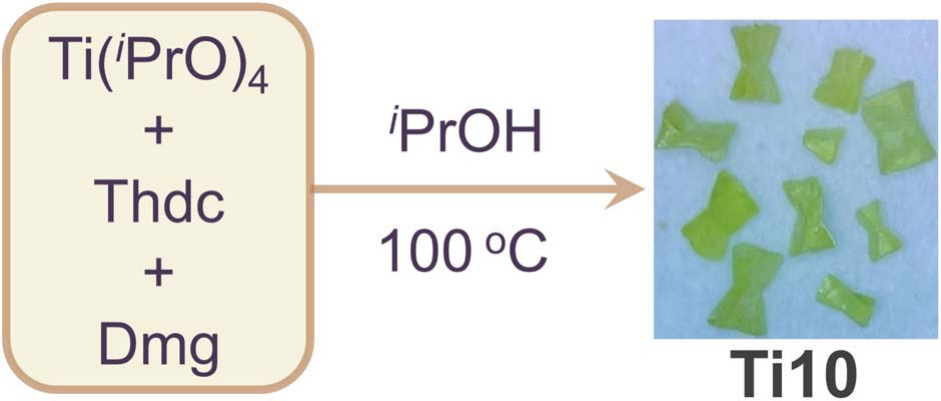
Schematic diagram of the synthesis for Ti10 (Thdc = 2,5-thiophenedicarboxylic acid; Dmg = dimethylglyoxime; ^i^PrOH = isopropanol).

**Fig. 1:**
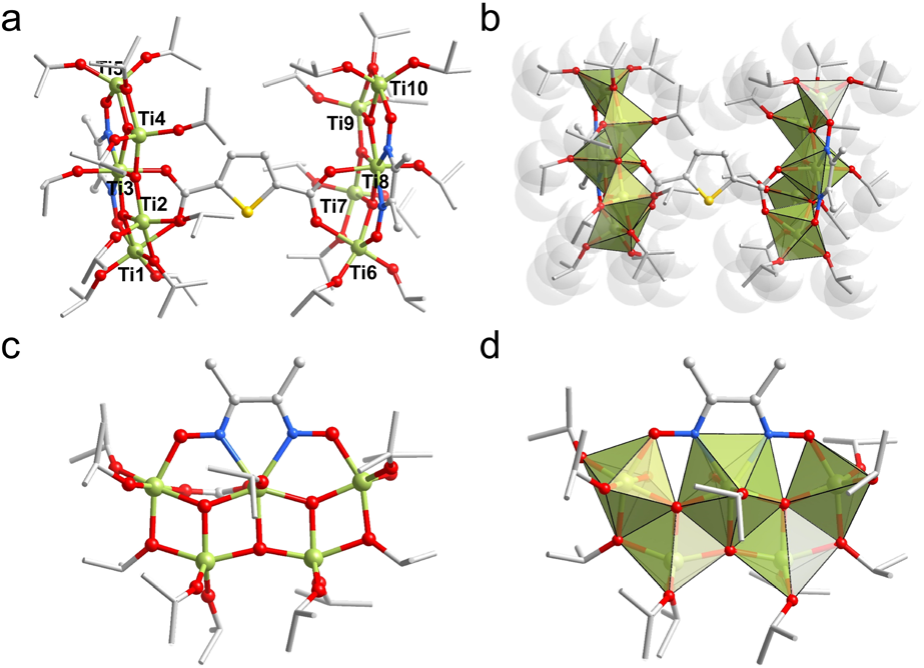
(a) Ball-stick view of Ti10. (b) Polyhedral view of Ti10. (c) Ball-stick view of {Ti_5_Dmg} layer. (d) Polyhedral view of {Ti_5_Dmg} layer. Color code: light green, Ti; yellow, S; red, O; gray, C; blue, N.

Ti10 was synthesized *via* a solvothermal reaction of Ti(^i^PrO)_4_, Dmg, and Thdc ligands dissolved in isopropanol and reacted at 100 °C for three days (Scheme 1). After cooling to room temperature, the mixture was left to stand for one week, resulting in the formation of yellow crystals with a moderate yield (Fig. S1†). It is well-known that solvents play a crucial role in influencing the structure of metal clusters. Therefore, we have performed numerous parallel experiments in the synthesis of Ti10, changing the solvents, such as ethanol or acetonitrile, while keeping all other synthesis variables constant. However, no other crystalline products could be isolated, suggesting the irreplaceable role of isopropanol in the formation of Ti10. Single crystal X-ray diffraction (SCXRD) analysis revealed that Ti10 crystallizes in the monoclinic system with the *P*1̄ space group. Its asymmetric unit contains one complete cluster. The overall structure of Ti10 appears dumbbell-shaped, with the Thdc ligand serving as the horizontal bar, and the {Ti_5_Dmg} layers on each side acting as the weight plates. Ti10 consists of 10 Ti atoms, 6 μ_3_-O atoms, 1 Thdc ligand, 2 Dmg ligands, and 22 isopropanol molecules (Fig. 1a). This structure is similar to the PTC-211 previously reported by Zhang.^40^ The main difference is that we used sulfur-containing heterocyclic Thdc ligand, while they used linear ligands terephthalic acid (Fig. S2†). Given that these clusters share a highly similar titanium-oxo core and are connected by various organic ligands, they could function as a model system for a comprehensive examination of the variations in properties resulting from distinct ligands.

Interestingly, the ten Ti atoms exhibit three different coordination environments (5-, 6-, and 7-coordinated) (Fig. 1b). Specifically, Ti3 and Ti8 are seven-coordinated, each coordinating with three μ_3_-O atoms, two N atoms from Dmg, one O atom from Thdc, and one O atom from isopropanol molecule, forming a pentagonal bipyramidal coordination environment. Ti1 and Ti6 are six-coordinated, with each coordinating with one μ_3_-O atom, one O atom from Dmg, one O atom from Thdc, and three O atoms from different isopropanol molecules, forming slightly distorted octahedral coordination environments. However, the remaining Ti atoms (Ti2, Ti4, Ti5, Ti7, Ti9, and Ti10) are five-coordinated, displaying a trigonal bipyramidal coordination environment.

Structurally, one Dmg ligand connects Ti1, Ti3, and Ti5 atoms in a μ_3_-η_O_^1^:η_N_^1^:η_N_^1^:η_O_^1^ coordination mode (Ti–N = 2.25–2.26 Å, Ti–O = 1.95–1.99 Å), forming the {Ti_3_Dmg} unit, which is further connected to two additional Ti atoms (Ti2 and Ti4) through three μ_3_-O atoms, resulting in the {Ti_5_Dmg} layer (Fig. 1c and d). One Thdc ligand acts as a linker to connect two {Ti_5_Dmg} units by its carboxyl groups (Ti–O = 2.07–2.23 Å), forming the final {Ti_10_} metal framework. The periphery of {Ti_10_} is further shielded by 22 isopropanol molecules, with 18 of them acting as monodentate ligands and 4 as bridging ligands, with Ti–O bond lengths ranging from 1.76 to 2.04 Å. It's worth noting that Ti10 could potentially serve as an effective catalyst, as the coordinating solvent molecules in this structure can readily dissociate during catalysis, forming the corresponding active sites to facilitate catalytic reactions.^41^

### General characterization of Ti10

The experimental powder X-ray diffraction (PXRD) pattern of Ti10 was well-matched with the simulated patterns derived from SCXRD data, providing evidence of high phase purity (Fig. S3†). Thermal stability assessment of Ti10 was carried out through thermogravimetric analysis (TGA), and the corresponding results indicate its high structural integrity was maintained at temperatures below 190 °C (Fig. S4†). Infrared (IR) spectroscopy of Ti10 shows that the peaks at 2974, 2934, and 2862 cm^−1^ can be assigned to *ν*(C–H) stretching vibrations of isopropoxide groups. The vibrations at around 1527 cm^−1^ indicate the coordination vibrations of carboxyl groups of Thdc. The band at ∼1370 cm^−1^ represents the stretching vibrations of *ν*(C=N) of Dmg (Fig. S5†). Energy-dispersive X-ray spectroscopy (EDS) analysis and mapping for Ti10 were presented to determine the chemical composition (Fig. S6 and S7†). Additionally, X-ray photoelectron spectroscopy (XPS) reveals that the titanium elements within Ti10 are found in the Ti(iv) oxidation state (Fig. S8†). This result is consistent with the BVS analysis (Table S1†).

### ESI-MS of Ti10

**Fig. 2:**
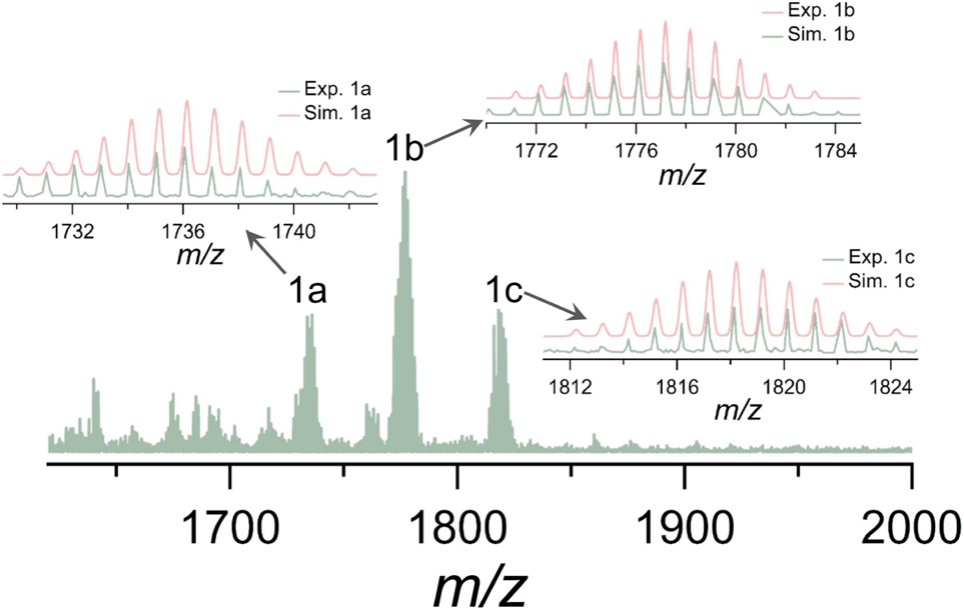
Positive-ion mode ESI-MS of Ti10 dissolved in CH_2_Cl_2_. Insets show the experimental (light green) and simulated (light coral) isotopic distribution patterns for species 1a–1c.

Electrospray ionization mass spectrometry (ESI-MS) serves as a supplementary method to X-ray crystallography when studying clusters, offering valuable information about the chemical composition and charge state of metal nanoclusters in solution.^42–45^ To elucidate the solution behavior of Ti10, ESI-MS was performed in positive ion mode after Ti10 was dissolved in CH_2_Cl_2_. As illustrated in Fig. 2, the ESI-MS spectrum exhibits a collection of signals with the most prominent peak, 1b, at *m*/*z* = 1777.1115. This peak can be attributed to [Ti_10_O_6_(Thdc)(Dmg)_2_(^i^PrO)_13_(H_2_O)_2_]^+^ (calcd *m*/*z* = 1777.1793), which corresponds to the loss of nine ^i^PrO^−^ molecules from Ti10. Some other relatively weak satellite peaks, 1a and 1c, are centered at *m*/*z* = 1736.0641 and *m*/*z* = 1818.1487, respectively. These peaks are assigned to [Ti_10_O_6_(Thdc)(Dmg)_2_(^i^PrO)_12_(H_2_O)_3_]^+^ (calcd *m*/*z* = 1736.1356) and [Ti_10_O_6_(Thdc)(Dmg)_2_(^i^PrO)_14_(H_2_O)]^+^ (calcd *m*/*z* = 1818.2186), respectively. All of these species (1c to 1a) can be considered as the result of the sequential loss of ^i^PrO^−^ molecules. The results reveal that fragmentation occurred during the ESI process, but the integrity of the metal skeleton is maintained. The loss of ^i^PrO^−^ molecules during ionization is attributed to the fact that they are weakly bound to the cluster surface compared to Dmg and Thdc ligands.

### Photoelectric properties

**Fig. 3:**
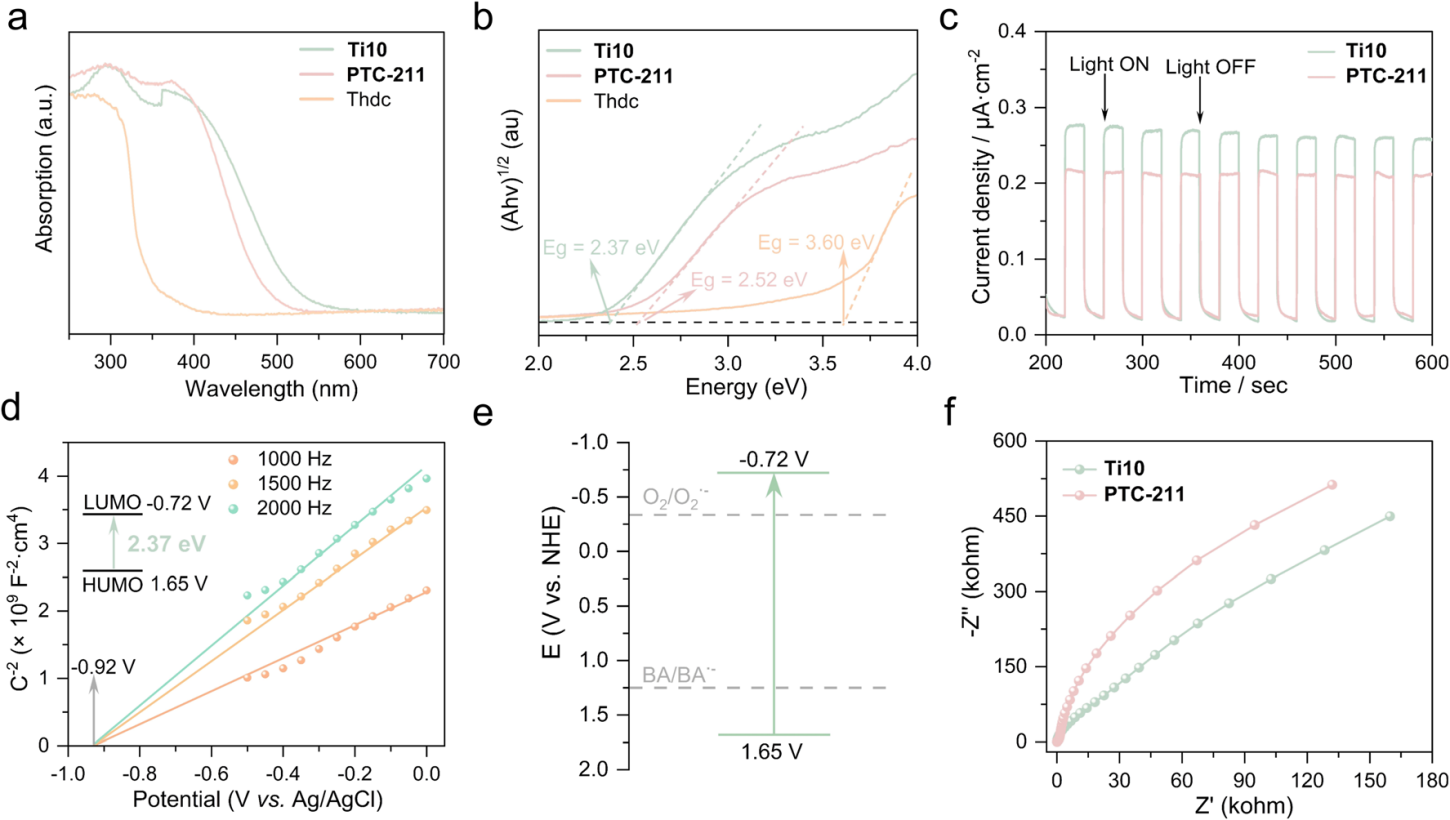
(a) Solid-state UV-visible absorption spectrum of Ti10, PTC-211, and Thdc. (b) Tauc plots of Ti10, PTC-211, and Thdc. (c) Transient photocurrent responses of Ti10 and PTC-211 under Xe lamp irradiation. (d) Mott–Schottky (M–S) plots of Ti10 modified electrode at different frequencies. (e) Energy band diagram of Ti10. (f) Electrochemical impedance spectroscopy (EIS) Nyquist plots of Ti10 and PTC-211.

Diffuse reflectance spectroscopy analysis was employed to examine the UV-vis absorption of Ti10. Furthermore, we selected PTC-211 as a research representative because the structures of Ti10 and PTC-211 are isomorphic. This allows us to better study the property differences caused by different ligands. As illustrated in Fig. 3a, the solid-state UV-visible absorption spectra show that the absorption bands of Ti10, PTC-211, and Thdc can extend to 538, 498, and 369 nm, respectively. Ti10 exhibited a broader absorption than PTC-211, indicating that Thdc ligand influences the light absorption capability of TOCs. Furthermore, Ti10 also demonstrated superior visible light absorption compared to the free Thdc ligand. As is known, the light absorption of titanium-oxo clusters with a relatively large band gap is primarily derived from O → Ti charge transfer transitions.^46^ Therefore, the broader absorption of Ti10 should be mainly attributed to the co-coordination of Thdc and Dmg. Based on the Kubelka–Munk function of (*αhυ*)^1/2^ = *κ*(*hυ* − *E*_g_) (*E*_g_ is the band gap (eV), *h* is the Planck's constant (J s), *ν* is the light frequency (s^−1^), *κ* is the absorption constant and *α* is the absorption coefficient), the optical band gaps of Ti10, PTC-211, and Thdc were estimated to be 2.37, 2.52, and 3.60 eV (Fig. 3b), respectively.^47^

The transient photocurrent response assessments were conducted to evaluate the capabilities of these TOCs in separating photogenerated electron–hole pairs. The photocurrent response experiments were carried out in a three-electrode cell, with TOC-coated ITO glass as the working electrode, Ag/AgCl as the reference electrode, and platinum wire as the counter electrode. All experiments were carried out in a 0.2 mol L^−1^ Na_2_SO_4_ electrolyte solution, under the illumination of a 150 W xenon light source, with on–off cycling intervals of 20 s. As shown in Fig. 3c, a consistent and reproducible photocurrent response was observed when the Xe lamp was switched on and off, indicating their good photoelectric response and high stability. Remarkably, the transient photocurrent density of the Ti10 electrode (0.25 μA cm^−2^) was higher than that of the PTC-211 electrode (0.20 μA cm^−2^). This suggests that Ti10 has better photogenerated electron separation and transfer capabilities due to the introduction of Thdc ligand.

Mott–Schottky measurements at frequencies of 1000, 1500, and 2000 Hz were performed to determine the flat-band potential of Ti10. As shown in Fig. 3d, the Mott–Schottky plot of Ti10 exhibits a positive slope, confirming its n-type semiconductor-like characteristic.^48^ The lowest unoccupied molecular orbital (LUMO) position of Ti10 was determined to be −0.72 V (*vs.* NHE, pH = 7). Combined with the band gap obtained from UV–Vis diffuse reflectance spectra and LUMO, the highest occupied molecular orbital (HOMO) position of Ti10 was evaluated to be 1.65 V (*vs.* NHE). Evidently, Ti10 exhibits a significantly higher HOMO value, rendering it suitable for potential applications in diverse photoinduced organic synthesis reactions, including amine oxidation. Additionally, the adequate band structure holds significant importance for catalysts involved in photocatalytic reactions. It is apparent that the LUMO value of Ti10 is more negative than the theoretical potential for the reduction of O_2_ to superoxide radical (−0.33 V *vs.* NHE), a key active species in organic oxidation reactions.^49^ This implies that Ti10 possesses theoretical viability for the photocatalytic transformation of O_2_ into superoxide radical anions (O_2_˙^−^) intermediates (Fig. 3e). Additionally, Ti10 demonstrates a faster interfacial charge transfer process than PTC-211, as evidenced by electrochemical impedance spectroscopy (EIS) Nyquist plots (Fig. 3f).

### Photocatalytic oxidative coupling reaction of benzylamine

Due to the outstanding photophysical characteristics outlined earlier, we carried out the photocatalytic oxidative coupling of BA. Through systematic optimization, we found that when subjecting 22 mg of Ti10 and 0.2 mmol of BA in 4 mL of CH_3_CN to 410 nm LED irradiation under O_2_ at room temperature for 18 hours, Ti10 exhibited remarkable catalytic activity. The conversion efficiency reached an impressive 99%, accompanied by a selectivity of approximately 99% for *N*-benzylidenebenzylamine (BDA), which is comparable to the conversion rate of amines in most reported Ti-related materials (Table S6†).

**Fig. 4:**
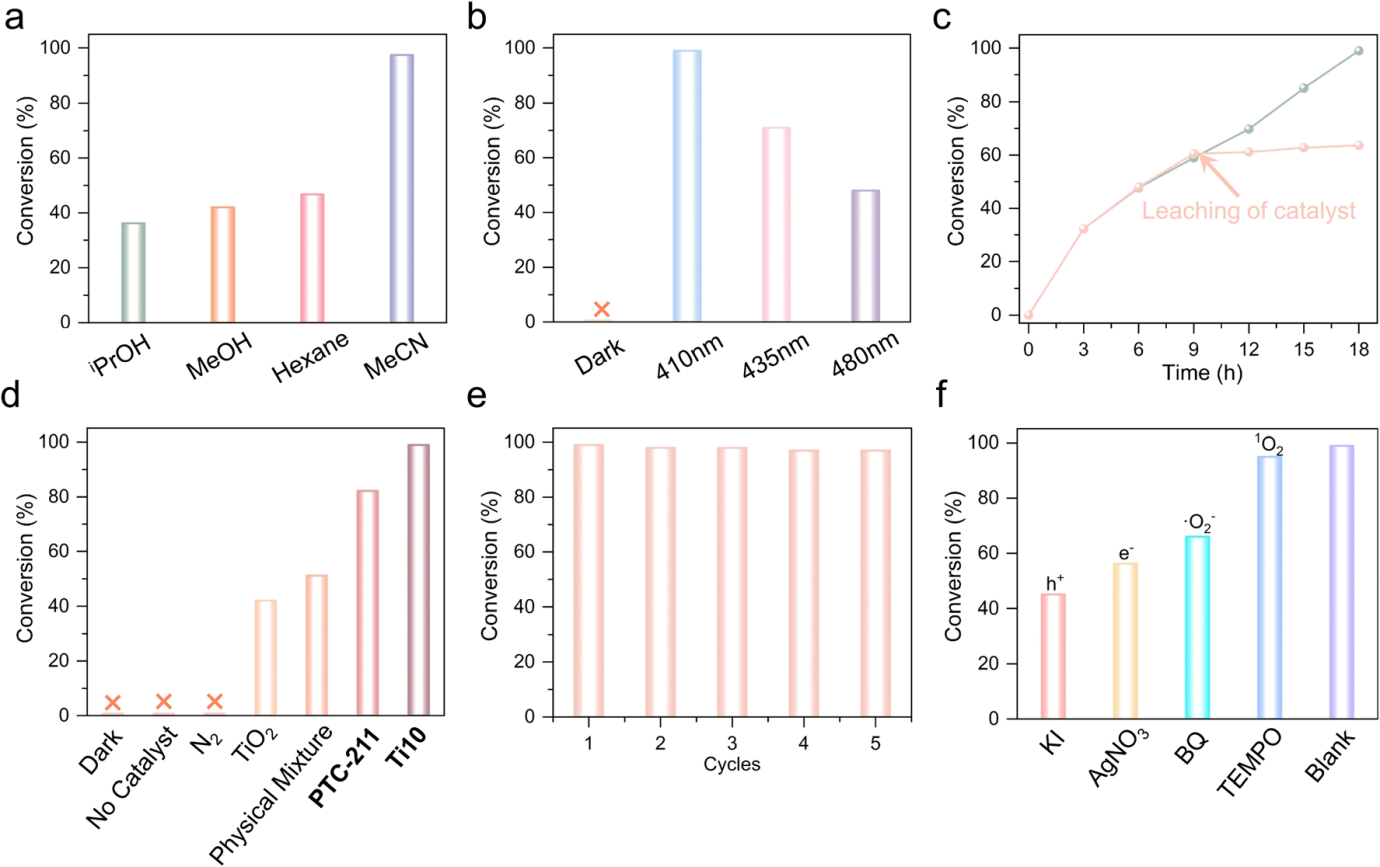
(a) The effects of various solvents on the reaction. (b) The effects of different photosources on the reaction. (c) Time profile for the reaction. (d) Photocatalytic performance of benzylamine conversion under different reaction conditions. (e) Catalytic durability of Ti10. (f) Photocatalytic conversion of benzylamine in the presence of radical scavengers (KI, AgNO_3_, BQ, and TEMPO).

Interestingly, the choice of solvent significantly impacts the reaction (Fig. 4a and Table S2†). While CH_3_CN promotes the generation of BDA, the employment of isopropanol, methanol, and *n*-hexane as solvents in the performance evaluations resulted in a marked decline in BA conversion, highlighting their significant influence on the reaction rate. Nevertheless, the type of solvent does not exert a noteworthy influence on product selectivity.

Additionally, the reaction is significantly influenced by the photosource. As depicted in Fig. 4b, the impact of photosources with different wavelengths on the reaction was examined (Table S3†). The subsequent optimization results indicated that the 410 nm LED outperformed other photosources (435 nm and 480 nm LEDs), leading to the production of the target product BDA with a favorable yield and selectivity. Subsequently, Ti10 served as the catalyst, CH_3_CN as the solvent, and a 410 nm LED as the light source during the time screening process, leading to a 99% yield of BDA in 18 hours (Fig. 4c). The leaching of the catalyst in the experiment demonstrated the heterogeneous catalytic characteristics.

Fig. 4d illustrates the photocatalytic oxidative coupling of BA under various conditions. The results of the blank experiment indicated the indispensability of Ti10 and O_2_, as no product was detected in the absence of catalyst or O_2_. We have also performed the reaction directly in the air. The conversion efficiency is lower than normal (under an oxygen atmosphere), demonstrating the necessity of O_2_ (Table S4,† entry 12). Additionally, under the dark condition, no product was detected, providing further evidence that the BA oxidation is facilitated by a photocatalytic process (Table S4,† entries 1–3). Moreover, a comparative experiment was conducted to assess the performance of TiO_2_ and the physical mixture (TiO_2_, Thdc, and Dmg). It is evident that TiO_2_ and the physical mixture exhibit inferior catalytic performance, achieving conversions of 42% and 51%, respectively (Table S4,† entries 4 and 5). This underscores the positive influence of the binding between ligands and metals in facilitating the reaction. Additionally, the BA conversion rate of Ti10 surpasses that of PTC-211, providing evidence that the introduction of the Thdc ligand significantly impacts the photocatalytic performance (Table S4,† entries 6 and 7).

Moreover, the cycling stability of Ti10 in the photocatalytic oxidative coupling of BA was examined (Fig. 4e). The results indicated that, after 5 reaction cycles, the conversion rate exhibited a minor decrease, suggesting satisfactory cycling performance. Besides, the IR spectra of the recovered catalyst aligned with those of the fresh Ti10 catalyst after five cycles of reuse (Fig. S9†).

In order to explore the application scope of Ti10, we conducted photocatalytic oxidative coupling reactions using various amine substrates. These substrates included benzylamines with diverse substituted groups (–F, –Cl, –Br, –CH_3_, –OCH_3_, –C(CH_3_)_3_, and –CF_3_ groups), along with 2-thiophenemethylamine (Table S5 and Fig. S10–S20†). Notably, all these substrates displayed high conversion rates, resulting in the formation of their respective imines.

In order to explore the mechanism of photocatalytic oxidative coupling of amine compounds, a series of controlled experiments were conducted (Fig. 4f). By introducing the electron scavenger AgNO_3_ and the hole scavenger KI, the conversion rate was lowered to 56% and 45%, respectively. This highlights the affirmative contribution of photogenerated electrons and holes to this reaction. Furthermore, benzoquinone (BQ) and 2,2,6,6-tetramethylpiperidin-1-oxyl (TEMPO) were introduced into the mixture to act as scavengers for superoxide radicals (˙O_2_^−^) and singlet oxygen (^1^O_2_), respectively. The findings indicate the significant contribution of ˙O_2_^−^ as a primary reactive oxygen species in the synthesis of imines, which is similar to reported examples.^50^ Typically, photogenerated electrons drive the formation of reactive species ˙O_2_^−^ and ^1^O2 by initiating the activation of O_2_.^51^

**Fig. 5:**
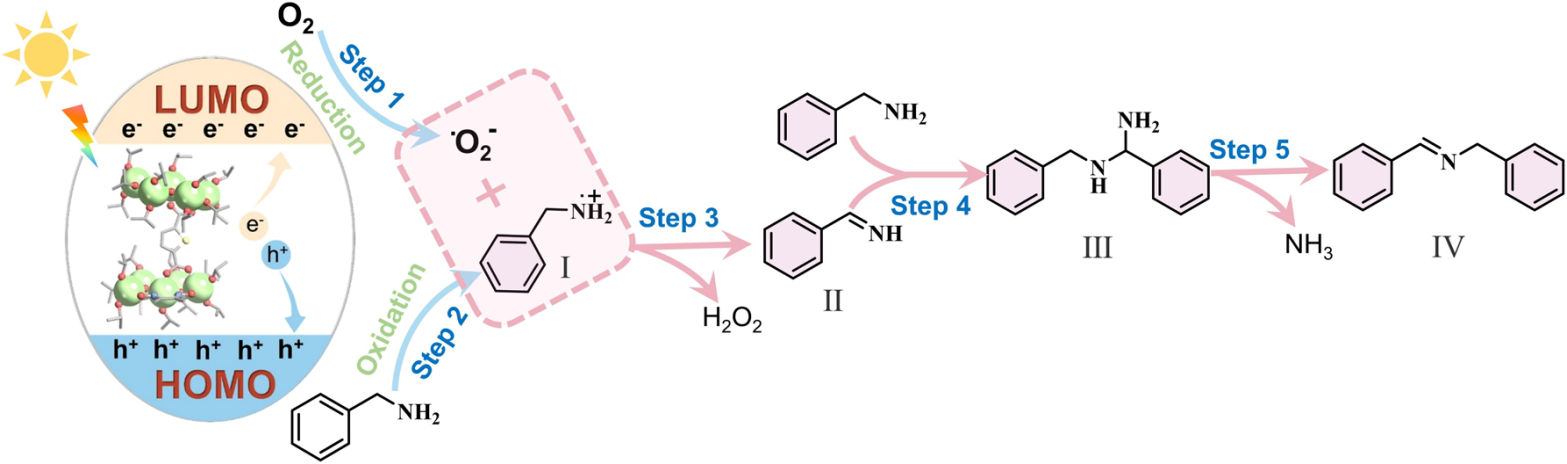
Proposed charge transfer process for the photocatalytic oxidative coupling of benzylamine over Ti10.

Based on both the experimental results and relevant literature,^28,29,48,50,52,53^ we propose a plausible mechanism for the photocatalytic oxidative coupling of BA. Upon irradiation, as depicted in Fig. 5, the Ti10 photocatalyst undergoes excitation, leading to the formation of electron–hole pairs. Photogenerated holes oxidized adsorbed benzylamine molecules, forming benzylamine radical cations (I), while photogenerated electrons reduced molecular oxygen, generating superoxide radicals (˙O_2_^−^). Following this sequence, intermediate I underwent a reaction with ˙O_2_^−^, giving rise to the generation of intermediate II. Subsequently, intermediate II is susceptible to attack by another free benzylamine molecule, ultimately leading to the formation of aminal (III). Finally, subsequent to the release of ammonia, the ultimate product, *N*-benzylidenebenzylamine (IV), was synthesized.

## Conclusions

In summary, we have successfully constructed a dumbbell-shaped crystalline titanium oxo cluster, Ti10, through a facial one-pot solvothermal strategy, and treated it as a catalyst for the photocatalytic oxidative coupling of amines. In this structure, Thdc serves as the horizontal bar, while the {Ti_5_Dmg} layers on each side act as the weight plates. Ti10 broadens its light absorption range into the visible spectrum as a result of the coordinated presence of Thdc and Dmg ligands. In contrast to PTC-211, the introduction of the Thdc ligand significantly improves the charge transfer within the Ti10 structure. On the basis of these advantages, Ti10 and PTC-211 acted as photocatalysts to conduct the photocatalytic oxidative coupling reaction of BA. The Ti10 exhibits superior catalytic performance compared to PTC-211. Our study serves as a significant example of the rational design of TOC-based photocatalysts with more precise functionalities to achieve effective photocatalytic conversion of organic compounds.

## Conflicts of interest

There are no conflicts to declare.

## Supplementary Material

RA-014-D4RA00117F-s001

RA-014-D4RA00117F-s002
