# Supplementary material for: Accurate assembly of thiophene-bridged titanium-oxo clusters with photocatalytic amine oxidation activity
Source: RSC Adv. 2024 Mar 6;14(11):7924–31. doi: 10.1039/d4ra00117f (PMC10915587; doi:10.1039/d4ra00117f)
Supplement: RA-014-D4RA00117F-s001 [file RA-014-D4RA00117F-s001.pdf]

Electronic Supplementary Material (ESI) for RSC Advances.
This journal is © The Royal Society of Chemistry 2024

Electronic Supporting Information

Accurate assembly of thiophene-bridged titanium-oxo clusters with photocatalytic amine oxidation activity

Haoran Nai,‡[a] Jinle Hou,*‡[a] Jinyu Li,[a] Xiaoxi Ma,[a] Yujia Yang,[a] Konggang Qu,[a] Xianqiang Huang*[a] and Lianzhi Li*[a]

[a] Shandong Provincial Key Laboratory of Chemical Energy Storage and Novel Cell Technology, and School of Chemistry and Chemical Engineering, Liaocheng University, Liaocheng, 252000, People's Republic of China. E-mail: houjinle@lcu.edu.cn (J. L. Hou); hxq@lcu.edu.cn (X. Q. Huang); lilianzhi1963@163.com (L. Z. Li).

‡ These authors contributed equally to this work.

Experimental Procedures

Materials and Instruments

All solvents and reagents were commercially available, analytical grade and used without further purification. 2, 5-thiophenedicarboxylic acid (Thdc), dimethylglyoxime (Dmg) were purchased from Macklin. Ti(^{i}PrO)$_4$ was purchased from Adamas and isopropanol was purchased from Sinopharm. Benzylamine (BA) and its extended substrates were purchased from Adamas.

Powder X-ray diffraction (PXRD) data were carried out on a microcrystalline powdered sample using a Rigaku SmartLab-9Kw diffractometer using Cu radiation (λ = 1.54184 Å). Thermogravimetry (TG) analysis was performed on a STA449F5/QMS403D instrument (Mettler-Toledo, Schwerzenbach, Switzerland) with a heating rate of 10 °C min^{-1} from 20 to 800 °C in N_2 flow. The solid-state UV/Vis spectra data of the cluster samples were obtained using a Carry 500 UV-VIS spectrophotometer with scanning wavelength range from 200 nm to 800 nm. Fourier transform infrared spectroscopy (FTIR) measurements were obtained using a Nicolet iS50 spectrophotometer. The X-ray photoelectron spectroscopy (XPS) spectra were collected on a Thermo Scientific ESCALAB Xi$^+$ instrument. The energy dispersive X-ray (EDX) spectra and element mapping of samples were acquired on a Thermo Fisher Scientific FIB-SEM-GX4 scanning electron microscope. The high-resolution electrospray ionization mass spectrometry (ESI-MS) of **Ti10** were acquired on an ACQUITY UPLC I-Class/Xevo G2-XS QTOF (Waters, Milford, USA) mass spectrometer. The data analysis of the mass spectrum was performed based on the isotope distribution patterns using MassLynx Analysis software (Version 4.1). Electrochemical measurements were carried out on a CHI 660E electrochemical workstation. Gas chromatographic (GC) analysis was carried out on a Shimadzu GC-2014C instrument equipped with a flame ionization detector (FID). Gas chromatogram and mass spectrum authentication were performed on an Agilent 7000D

Synthesis

Synthesis of $Ti_{10}O_6(Thdc)(Dmg)_2(^iPrO)_{22}$ (Ti10)

$Ti(^iPrO)_4$ (0.92 mL, 3 mmol), 2,5-thiophenedicarboxylic acid (Thdc) (100 mg, 0.58 mmol), and dimethylglyoxime (Dmg) (69 mg, 0.59 mmol) in isopropanol (5 mL) were ultrasonically dissolved. The mixture was sealed in 20 mL glass vial and then heated at 100 °C for 72 h. After the mixture was cooled to room temperature for a week, yellow crystals were obtained by filtration and were fully washed several times with isopropanol. Yield: 50 mg. Elemental analysis calc. (found) for **Ti10** ($C_{80}H_{168}N_4O_{36}S_1Ti_{10}$): C, 42.27 (42.76); N, 2.46 (2.28); H, 7.45 (7.68)%.

The synthesis of **PTC-211** has been reported in the literature.[1]

Single-crystal X-ray diffraction

X-ray diffraction measurements for the **Ti10** were performed on a Rigaku XtaLAB Synergy diffractometer operating with Cu-Kα (λ = 1.54184 Å) at 213K. These structures were solved by the inherent phase method in the SHELXT program and refined by full-matrix least squares techniques against F^2 using the SHELXL program through the OLEX2 interface. Appropriate restraints or constraints were applied to the geometry and the atomic displacement parameters of the atoms in the cluster. The structure was examined using the Addsym subroutine of PLATON to ensure that no additional symmetry could be applied to the models. Pertinent crystallographic data collection and refinement parameters are collated in Table S6. Selected bond lengths and are collated in Table S7 and S8. The crystallographic data for **Ti10** was delivered to the Cambridge Crystallographic Data Centre (CCDC) with No. 2322179. These data can be obtained from the CCDC via www.ccdc.cam.ac.uk/data_request/cif.

Electrochemical measurements

Photoelectrochemical measurements

Photoelectrochemical measurements were carried out on CHI660E electrochemical workstation in a standard three-electrode electrochemical cell with a working electrode, a platinum plate as counter electrode and a saturated Ag/AgCl electrode as reference electrode. A sodium sulfate solution (0.2 M) was used as the electrolyte and use a 150W Xe lamp as the light source.

Preparation of the working electrode: 5 mg crystals powder was mixed with 0.99 mL ethanol and 10 μL Nafion D-520 dispersion solutions and sonicated for 30 minutes. Subsequently, 200 μL of slurry was transferred and coated on ITO glass plates (1 cm×2 cm) then dried at room temperature.

Mott-Schottky plot measurements

The Mott-Schottky plots were carried out on the electrochemical workstation via a conventional three-electrode system with a working electrode, a platinum plate as counter electrode and a saturated Ag/AgCl electrode as reference electrode in a 0.2 M Na_2SO_4 aqueous solution at different frequencies of 1000 Hz, 1500 Hz and 2000 Hz. The working electrode was prepared same as photoelectrochemical measurements.

Electrochemical impedance spectroscopy

Electrochemical impedance spectra (EIS) measurements were also carried out on CHI660E electrochemical workstation via a conventional three-electrode system with a working electrode, a platinum plate as counter electrode and a saturated Ag/AgCl electrode as reference electrode in a 0.2 M Na_2SO_4 aqueous solution over a frequency range of 100 kHz-0.01 Hz.

Photocatalytic oxidative coupling of benzylamines

Typically, 22 mg of photocatalyst was dispersed in 4 mL of CH_3CN containing 0.2 mmol of BA in a 10 mL quartz reactor. Subsequently, the mixture was bubbled with O_2 for 5 minutes and irradiated with 410 nm LED (Xi'an Wattecs Experimental Equipment Co., LTD., China). Simultaneously, it was stirred at 500 rpm at room temperature under a molecular oxygen atmosphere (1 atm., balloon) in a Wattecs Parallel Photocatalytic Reactor (WP-TEC-1020HSL) for an appropriate reaction time (monitored by GC). After reaction completion as checked by GC/MS, the catalyst was separated by centrifugation and washed with CH_3CN. Other benzylamine derivatives with the same concentration were conducted using a similar approach. The conversion of BA and selectivity for N-benzylidenebenzylamine (BDA) product, were calculated with the following Equation (1) and (2):

$$Conversion\ (\%) = \frac{C_0 - C_{BA}}{C_{BA}} \times 100\%$$

$$Selectivity\ (\%) = \frac{2 \times C_{BDA}}{C_0 - C_{BA}} \times 100\%$$

where C_0 is the initial concentration of BA; C_{BA} and C_{BDA} stand for the concentration of the residual BA and the corresponding product of BDA at a certain time after the catalytic reaction, respectively.

Figure S1. The image of **Ti10** under optical microscope.

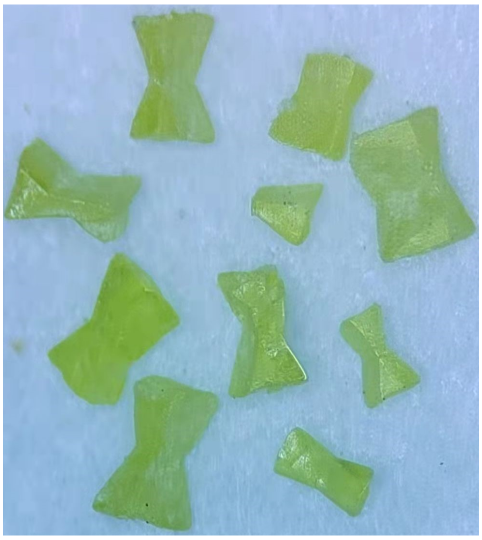

Figure S2. (a) Ball-and-stick view of the crystal structure of **Ti10**. (b) Ball-and-stick view of the crystal structure of **PTC-211**.

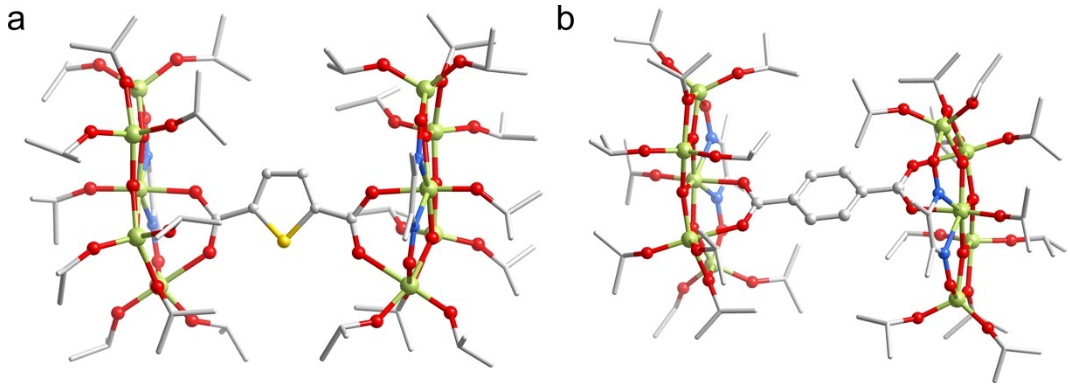

Figure S3. The compared PXRD patterns of **Ti10**.

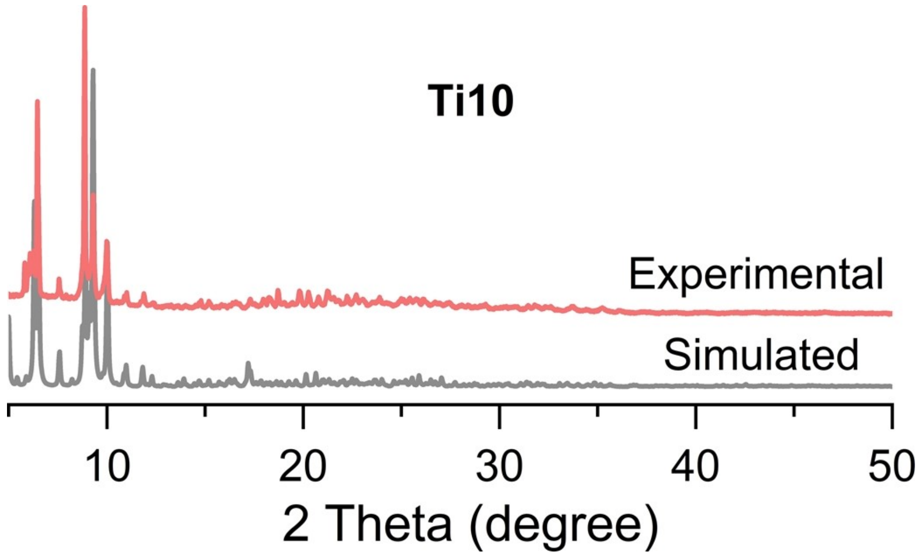

Figure S4. Thermogravimetry analysis (TGA) curve of **Ti10**.

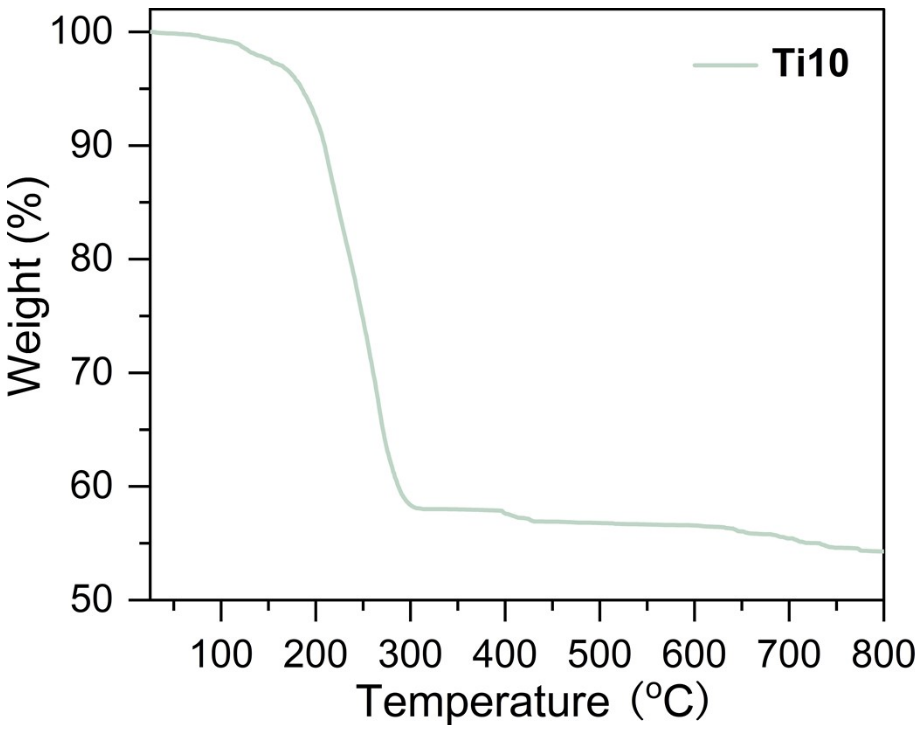

Figure S5. The Fourier transform infrared spectroscopy (FT-IR) of **Ti10**, Thdc and Dmg.

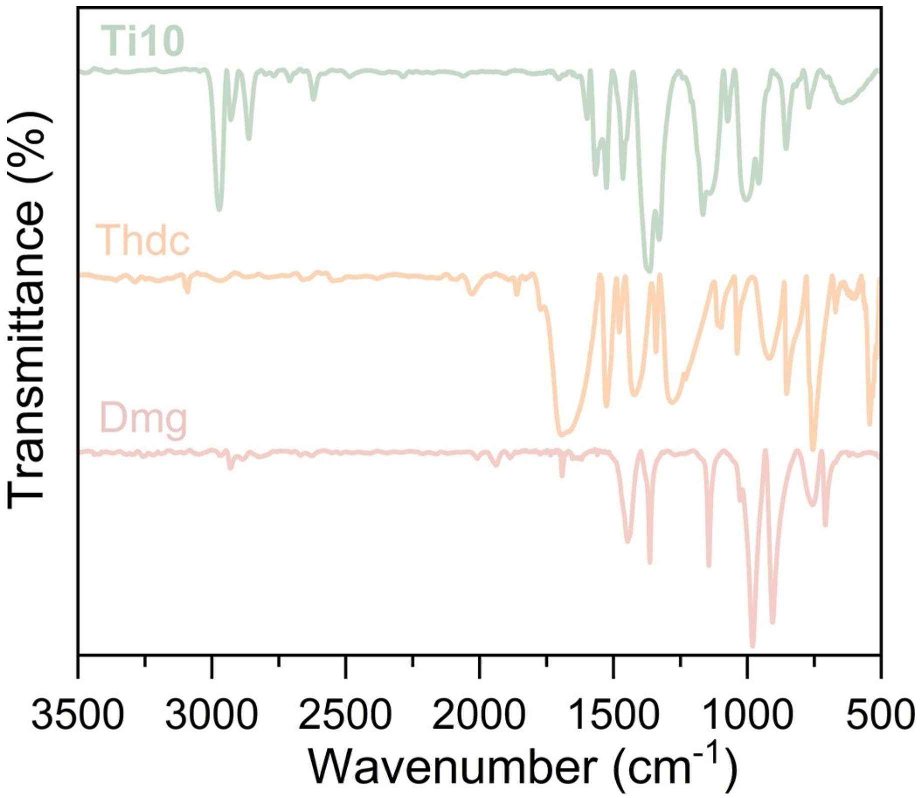

Figure S6. SEM and energy-dispersive X-ray spectroscopy of **Ti10**.

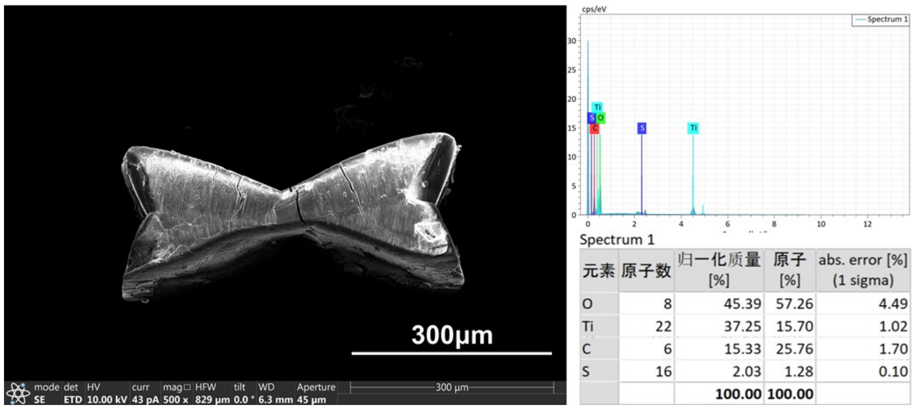

Spectrum 1

元素	原子数	归一化质量 [%]	原子 [%]	abs. error [%] (1 sigma)
O	8	45.39	57.26	4.49
Ti	22	37.25	15.70	1.02
C	6	15.33	25.76	1.70
S	16	2.03	1.28	0.10
		100.00	100.00	

Figure S7. Elemental mapping images of Ti, O and S of selected area for **Ti10**.

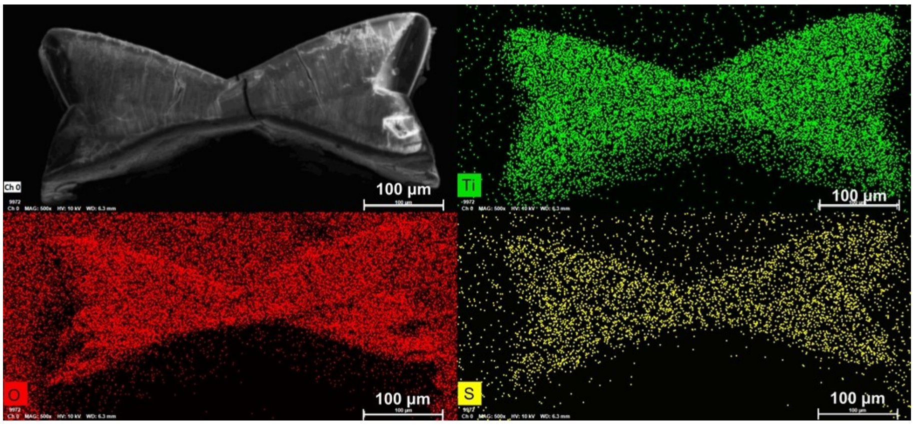

Figure S8. X-ray photoelectron spectroscopy (XPS) spectra of **Ti10**. XPS reveals that all Ti atoms in **Ti10** are exclusively in +4 oxidization state.

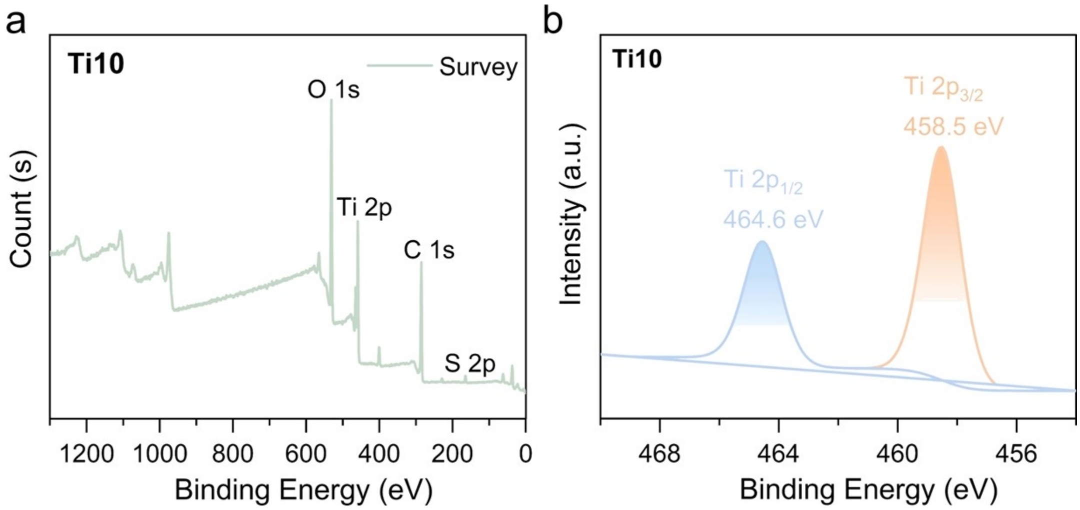

Figure S9. The IR spectra of **Ti10** before and after photocatalytic reaction, respectively.

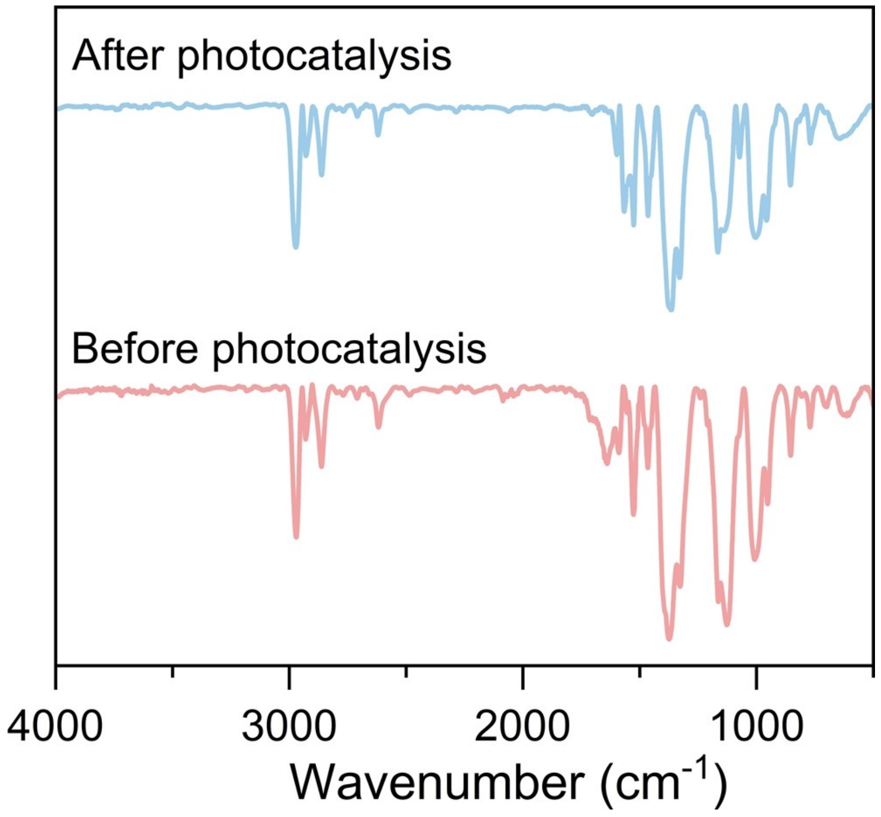

Table S1. Bond valence sum (BVS) analysis[a] for **Ti10**.

**Ti10**								
**Ti1 3.982**			**Ti2 4.039**			**Ti3 3.921**		
Ti1–O3	d=2.227	0.308	Ti2–O21	d=2.016	0.544	Ti3–O4	d=2.081	0.457
Ti1–O21	d=2.019	0.540	Ti2–O24	d=1.780	1.030	Ti3–O27	d=1.791	1.000
Ti1–O22	d=1.803	0.968	Ti2–O25	d=1.762	1.082	Ti3–O34	d=1.991	0.582
Ti1–O23	d=1.809	0.953	Ti2–O35	d=1.926	0.694	Ti3–O35	d=2.120	0.411
Ti1–O33	d=1.986	0.590	Ti2–O36	d=1.929	0.689	Ti3–O36	d=1.930	0.687
Ti1–O36	d=1.966	0.623				Ti3–N3	d=2.252	0.393
						Ti3–N4	d=2.253	0.391
**Ti4 4.119**			**Ti5 4.034**			**Ti6 4.001**		
Ti4–O26	d=1.781	1.027	Ti5–O28	d=2.005	0.561	Ti6–O1	d=2.219	0.315
Ti4–O28	d=2.035	0.517	Ti5–O30	d=1.768	1.064	Ti6–O6	d=1.989	0.586
Ti4–O29	d=1.753	1.108	Ti5–O31	d=1.788	1.008	Ti6–O7	d=1.968	0.620
Ti4–O34	d=1.922	0.702	Ti5–O32	d=1.954	0.644	Ti6–O17	d=1.797	0.984
Ti4–O35	d=1.890	0.765	Ti5–O34	d=1.894	0.757	Ti6–O18	d=2.014	0.547
						Ti6–O19	d=1.810	0.950
**Ti7 4.057**			**Ti8 3.908**					
Ti7–O7	d=1.921	0.704	Ti8–O2	d=2.078	0.460			
Ti7–O9	d=1.918	0.709	Ti8–O7	d=1.928	0.691			
Ti7–O16	d=1.761	1.084	Ti8–O8	d=1.970	0.616			
Ti7–O18	d=2.017	0.543	Ti8–O9	d=2.151	0.378			
Ti7–O20	d=1.785	1.016	Ti8–O15	d=1.797	0.984			
			Ti8–N1	d=2.259	0.385			
			Ti8–N2	d=2.251	0.394			
**Ti9 4.023**			**Ti10 3.997**					
Ti9–O8	d=1.939	0.670	Ti10–O5	d=1.956	0.640			
Ti9–O9	d=1.894	0.757	Ti10–O8	d=1.897	0.751			
Ti9–O10	d=1.783	1.022	Ti10–O11	d=1.774	1.047			
Ti9–O12	d=2.030	0.524	Ti10–O12	d=2.010	0.553			
Ti9–O14	d=1.773	1.050	Ti10–O13	d=1.789	1.005			

[a] $V_i = \Sigma S_{ij} = \Sigma \exp[(r_1 - r_{ij})/B]$, where r_{ij} is the bond length between atoms i and j (with r_1 = 1.791 for Ti^{iv}-O and 1.906 for Ti^{iv}-N). The constant B is referred to as the “universal parameter” and is approximately equal to 0.37 Å. S_{ij} is the valence of a bond between atoms i and j, and V_i is the sum of all bond valences of the bonds formed by a given atom.[2]

Table S2. Screening of solvents for photocatalytic benzylamine oxidation coupling over **Ti10**.[a]

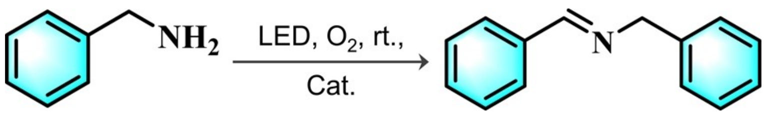

Entry	Solvent	Con. (%)[b]	Sel. (%)[b]
1	Methanol	42	97
2	Isopropanol	36	98
3	N-hexane	46	99
4	Acetonitrile	99	99

[a] BA (0.2 mmol), **Ti10** (22 mg), 4 mL solvent, reaction time 18 h under an O_2 atmosphere at room temperature, with a 410 nm LED. [b] Determined by GC.

Table S3. Photocatalytic oxidation of benzylamines using LED light sources with different wavelengths.[a]

Entry	Wavelength (nm)	Con. (%) [b]	Sel. (%) [b]
1	410	99	99
2	430	71	96
3	480	47	99

[a] BA (0.2 mmol), **Ti10** (22 mg), 4 mL CH_3CN, reaction time 18 h under an O_2 atmosphere at room temperature, using LEDs with different wavelengths. [b] Determined by GC.

Table S4. Optimization of the conditions for light driven oxidative coupling of benzylamines and control experiments to predict the mechanism.

Entry	Photocatalyst	Con. (%)[f]	Sel. (%)[f]
1[a]	No catalyst	-	-
2[a]	**Ti10** without light	-	-
3[a]	**Ti10**, N_2	-	-
4[a]	TiO_2	42	98
5[a]	TiO_2+Thdc+Dmg	51	98
6[a]	**Ti10**	99	99
7[a]	**PTC-211**	82	99
8[b]	**Ti10**	66	96
9[c]	**Ti10**	56	98
10[d]	**Ti10**	91	99
11[e]	**Ti10**	45	97
12[a]	**Ti10**, air	81	98

[a] BA (0.2 mmol), catalysts (22 mg), 4 mL CH_3CN, reaction time of 18 h under an O_2 atmosphere at room temperature, with a 410 nm LED. [b] For the same conditions as [a], but with benzoquinone (BQ) as a superoxide radicals ($^{\bullet}O_2^-$) scavenger. [c] Likewise, under identical conditions as [a], $AgNO_3$ was used as an electron scavenger. [d] Similarly, [a] was followed, but with 2,2,6,6-tetramethylpiperidin-1-oxyl (TEMPO) as a singlet oxygen ($^1O_2$) scavenger. [e] Following the conditions of [a], KI was used as a hole scavenger. [f] The results were determined by GC.

Table S5. Photocatalytic oxidative coupling reactions of various amines over the **Ti10** sample.

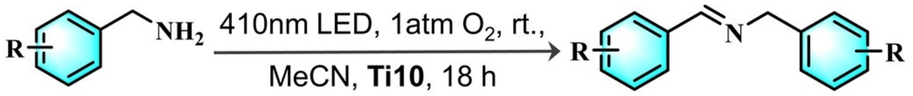

Entry	Substrate	Product	Con. (%)	Sel. (%)
1	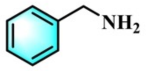	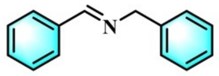	99	99
2	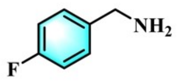	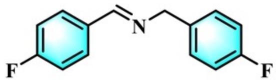	97	99
3	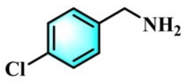	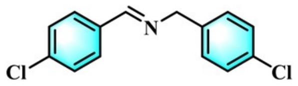	98	98
4	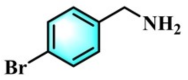	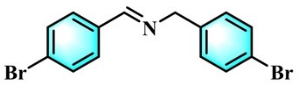	97	99
5	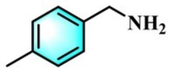	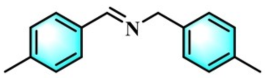	93	94
6	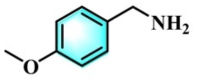	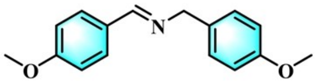	99	99
7	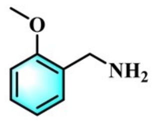	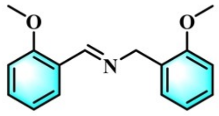	98	97
8	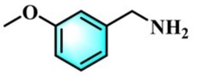	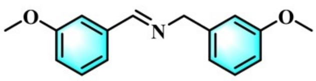	94	99
9	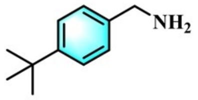	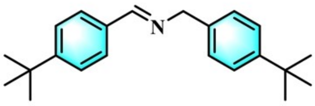	97	97
10	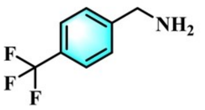	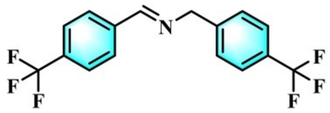	96	99
11	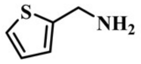	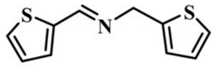	99	99

Table S6. Comparison results of **Ti10** with typical reported catalysts of some Ti-related materials.

Entry	Catalysts	Oxidant	Temperature	Time	Light source	Selectivity	Conversion	Ref.
1	**Ti-PMOF**	air	R.T.	30 min	623 nm LED	85 %	93 %	3
2	**PCN-222**	air	R.T.	1 h	$\lambda \geq 420$ nm	100 %	100 %	4
3	**ARS-TiO_2**	TEMPO	R.T.	2 h	blue LED	96 %	92 %	5
4	**NH_2-MIL-125(Ti)**	O_2	R.T.	12 h	$\lambda \geq 420$ nm	73 %	86 %	6
5	**Polyimide-TiO_2**	TEMPO	R.T.	50 min	460 nm LED	91 %	98 %	7
6	**TPA**	O_2	R.T.	8 h	$\lambda \geq 420$ nm	98 %	95 %	8
7	**Th6-C8A**	O_2	R.T.	16 h	$\lambda \geq 420$ nm	97 %	99 %	9
8	**Ti_5Fc**	TBHP	70 °C	8 h	$\lambda \geq 420$ nm	99 %	99 %	10
9	**$Ti_{11}Fc_2$**	TBHP	70 °C	8 h	$\lambda \geq 420$ nm	88 %	99 %	10
10	**$Ti_{22}Fc_4$**	TBHP	70 °C	8 h	$\lambda \geq 420$ nm	85 %	99 %	10
11	**$Ti_{13}L_6$**	H_2O_2	60°C	12 h	$\lambda \geq 420$ nm	99 %	99 %	11
**12**	**Ti10**	**O_2**	**R.T.**	**18 h**	**410 nm LED**	**99 %**	**99 %**	**This work**

TEMPO = 2,2,6,6-tetramethylpiperidin-1-yl)oxyl; TBHP = tert-butyl hydroperoxide; TPA = titanium-porphyrinic aerogels.

Table S7. Crystal data collection and sructure refinement for **Ti10**.

**Compound**	**Ti10**
Empirical formula	$C_{80}H_{168}N_4O_{36}STi_{10}$
Formula weight	2273.23
Temperature [K]	213.00(10)
Crystal system	triclinic
Space group (number)	$P\bar{1}$ (2)
a [Å]	16.5980(5)
b [Å]	14.186(2)
c [Å]	20.9945(7)
α [°]	74.614(3)
β [°]	75.671(3)
γ [°]	74.331(3)
Volume [Å^3]	5863.0(4)
Z	2
ρ_{calc} [g/cm^3]	1.287
μ [mm^{-1}]	6.239
F(000)	2398
Radiation	Cu K_{α} (λ=1.54184 Å)
2θ range [°]	5.09 to 134.16 (0.84 Å)
Index ranges	$-16 \leq h \leq 19$<br>$-22 \leq k \leq 22$<br>$-24 \leq l \leq 25$
Reflections collected	63279
Independent reflections	20665<br>R_{int} = 0.0560<br>R_{sigma} = 0.0551
Completeness to θ = 66.561°	98.7 %
Data / Restraints / Parameters	20665/691/1448
Goodness-of-fit on F^2	1.040
Final *R* indexes [$I \geq 2\sigma(I)$]	R_1 = 0.0448<br>wR_2 = 0.1198
Final *R* indexes [all data]	R_1 = 0.0796<br>wR_2 = 0.1774
Largest peak/hole [e/Å^3]	0.82/−0.82

Table S8. Selected bond lengths (Å) for **Ti10**.

**Ti10**			
**Atom-Atom**	**Length / Å**	**Atom-Atom**	**Length / Å**
Ti1–O3	2.227(3)	Ti6–O1	2.219(3)
Ti1–O21	2.019(3)	Ti6–O6	1.989(3)
Ti1–O22	1.803(3)	Ti6–O7	1.968(2)
Ti1–O23	1.809(3)	Ti6–O17	1.797(3)
Ti1–O33	1.986(3)	Ti6–O18	2.014(3)
Ti1–O36	1.966(3)	Ti6–O19	1.810(3)
Ti2–O21	2.016(3)	Ti7–O7	1.921(3)
Ti2–O24	1.780(4)	Ti7–O9	1.918(3)
Ti2–O25	1.762(3)	Ti7–O16	1.761(4)
Ti2–O35	1.926(3)	Ti7–O18	2.017(3)
Ti2–O36	1.929(3)	Ti7–O20	1.785(4)
Ti3–O4	2.081(2)	Ti8–O2	2.078(3)
Ti3–O27	1.791(3)	Ti8–O7	1.928(3)
Ti3–O34	1.991(3)	Ti8–O8	1.970(3)
Ti3–O35	2.120(3)	Ti8–O9	2.151(3)
Ti3–O36	1.930(3)	Ti8–O15	1.797(3)
Ti3–N3	2.252(3)	Ti8–N1	2.259(3)
Ti3–N4	2.253(3)	Ti8–N2	2.251(3)
Ti4–O26	1.781(5)	Ti9–O8	1.939(3)
Ti4–O28	2.035(3)	Ti9–O9	1.894(3)
Ti4–O29	1.753(4)	Ti9–O10	1.783(4)
Ti4–O34	1.922(3)	Ti9–O12	2.030(3)
Ti4–O35	1.890(3)	Ti9–O14	1.773(4)
Ti5–O28	2.005(3)	Ti10–O5	1.956(3)
Ti5–O30	1.768(4)	Ti10–O8	1.897(3)
Ti5–O31	1.788(3)	Ti10–O11	1.774(4)
Ti5–O32	1.954(3)	Ti10–O12	2.010(3)
Ti5–O34	1.894(3)	Ti10–O13	1.789(3)

Table S9. Selected band angles (º) of **Ti10**.

**Ti10**			
**Atom–Atom–Atom**	**Angle [°]**	**Atom–Atom–Atom**	**Angle [°]**
O21–Ti1–O3	82.62(11)	O36–Ti3–N4	142.61(12)
O22–Ti1–O3	82.83(12)	N3–Ti3–N4	67.90(11)
O22–Ti1–O21	105.54(13)	O26–Ti4–O28	91.71(19)
O22–Ti1–O23	97.09(14)	O26–Ti4–O34	131.4(2)
O22–Ti1–O36	163.27(12)	O26–Ti4–O35	97.35(19)
O23–Ti1–O3	179.09(13)	O29–Ti4–O26	111.6(3)
O23–Ti1–O21	96.54(13)	O29–Ti4–O28	98.35(19)
O23–Ti1–O33	95.76(13)	O29–Ti4–O34	116.1(2)
O23–Ti1–O36	99.57(12)	O29–Ti4–O35	105.85(18)
O33–Ti1–O3	85.16(11)	O34–Ti4–O28	72.88(12)
O33–Ti1–O21	154.89(12)	O35–Ti4–O28	148.75(13)
O36–Ti1–O3	80.54(10)	O35–Ti4–O34	78.79(12)
O36–Ti1–O21	74.17(11)	O30–Ti5–O28	99.93(19)
O36–Ti1–O33	82.25(11)	O30–Ti5–O31	107.39(19)
O24–Ti2–O21	91.64(16)	O30–Ti5–O32	100.35(18)
O24–Ti2–O35	96.64(15)	O30–Ti5–O34	120.66(17)
O24–Ti2–O36	139.05(19)	O31–Ti5–O28	97.53(16)
O25–Ti2–O21	102.41(15)	O31–Ti5–O32	92.62(15)
O25–Ti2–O24	108.4(2)	O31–Ti5–O34	131.93(15)
O25–Ti2–O35	105.23(16)	O32–Ti5–O28	153.42(13)
O25–Ti2–O36	112.23(18)	O34–Ti5–O28	74.13(12)
O35–Ti2–O21	146.85(12)	O34–Ti5–O32	80.80(11)
O35–Ti2–O36	77.83(11)	O6–Ti6–O1	85.12(11)
O36–Ti2–O21	75.00(11)	O6–Ti6–O18	154.81(11)
O27–Ti3–O4	172.72(13)	O7–Ti6–O6	81.93(10)
O27–Ti3–O34	97.26(13)	O7–Ti6–O18	74.18(11)
O27–Ti3–O35	97.85(12)	O17–Ti6–O1	178.86(14)
O27–Ti3–O36	92.42(12)	O17–Ti6–O6	95.96(14)
O27–Ti3–N3	92.35(13)	O17–Ti6–O7	99.53(12)
O27–Ti3–N4	86.74(13)	O17–Ti6–O18	95.77(14)
O35–Ti3–N3	146.80(11)	O17–Ti6–O19	97.12(13)
O35–Ti3–N4	143.87(12)	O18–Ti6–O1	83.37(12)
O36–Ti3–O4	87.58(10)	O19–Ti6–O1	82.41(11)
O36–Ti3–O34	144.92(12)	O19–Ti6–O6	95.04(12)
O36–Ti3–O35	73.28(11)	O19–Ti6–O7	163.30(12)
O36–Ti3–N3	74.79(11)	O19–Ti6–O18	105.52(13)

Continue above

Ti10			
**Atom–Atom–Atom**	**Angle [°]**	**Atom–Atom–Atom**	**Angle [°]**
O7–Ti7–O18	75.11(11)	O15–Ti8–O9	98.07(12)
O9–Ti7–O7	78.60(11)	O15–Ti8–N1	91.90(12)
O9–Ti7–O18	146.55(12)	O15–Ti8–N2	86.97(12)
O16–Ti7–O7	110.89(18)	N2–Ti8–N1	67.88(11)
O16–Ti7–O9	105.29(16)	O8–Ti9–O12	72.61(12)
O16–Ti7–O18	103.18(16)	O9–Ti9–O8	78.06(11)
O16–Ti7–O20	110.2(2)	O9–Ti9–O12	147.19(13)
O20–Ti7–O7	138.52(19)	O10–Ti9–O8	114.69(17)
O20–Ti7–O9	95.96(16)	O10–Ti9–O9	106.89(16)
O20–Ti7–O18	90.33(17)	O10–Ti9–O12	98.59(17)
O2–Ti8–O9	88.74(11)	O14–Ti9–O8	135.95(19)
O2–Ti8–N1	80.74(11)	O14–Ti9–O9	98.35(16)
O2–Ti8–N2	89.24(11)	O14–Ti9–O10	108.4(2)
O7–Ti8–O2	87.36(10)	O14–Ti9–O12	92.71(16)
O7–Ti8–O8	144.12(11)	O5–Ti10–O12	152.19(13)
O7–Ti8–O9	72.93(11)	O8–Ti10–O5	81.35(11)
O7–Ti8–N1	74.87(11)	O8–Ti10–O12	73.92(12)
O7–Ti8–N2	142.65(11)	O11–Ti10–O5	100.16(16)
O8–Ti8–O2	86.76(11)	O11–Ti10–O8	116.84(17)
O8–Ti8–O9	71.58(11)	O11–Ti10–O12	102.22(17)
O8–Ti8–N1	138.53(12)	O11–Ti10–O13	107.1(2)
O8–Ti8–N2	72.60(11)	O13–Ti10–O5	91.43(15)
O9–Ti8–N1	146.49(11)	O13–Ti10–O8	136.06(17)
O9–Ti8–N2	144.18(11)	O13–Ti10–O12	97.52(16)
O15–Ti8–O2	172.56(12)	C3–S1–C4	91.35(18)
O15–Ti8–O7	91.77(12)	O1–C5–O2	126.9(3)
O15–Ti8–O8	98.20(12)	O3–C6–O4	126.6(3)

Figure S10. Gas chromatogram and mass spectrum authentication of N-benzylideneaniline obtained from the GC-MS analysis after 18 h reaction over the **Ti10** sample.

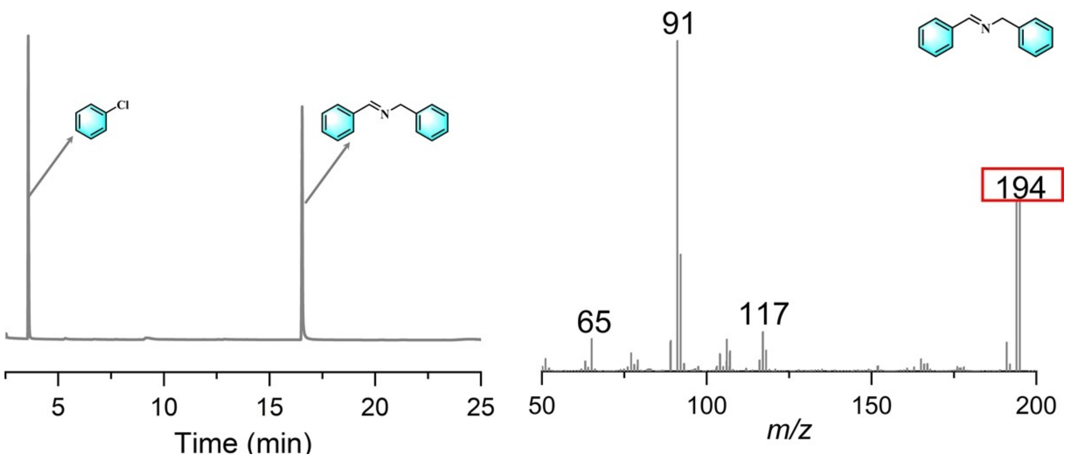

Figure S11. Gas chromatogram and mass spectrum authentication of N-(4-fluorobenzyl)-1-(4-fluorophenyl) methanimine obtained from the GC-MS analysis after 18 h reaction over the **Ti10** sample.

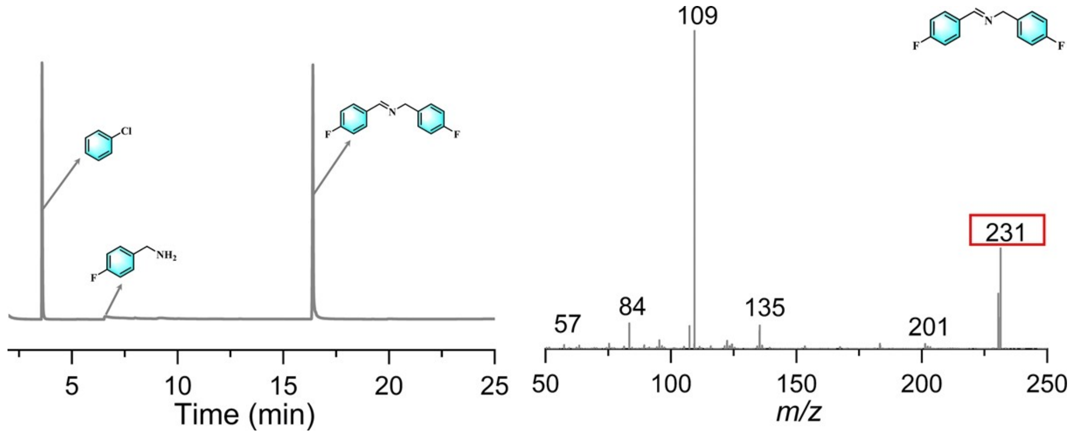

Figure S12. Gas chromatogram and mass spectrum authentication of N-(4-chlorobenzyl)-1-(4-chlorophenyl) methanimine obtained from the GC-MS analysis after 18 h reaction over the **Ti10** sample.

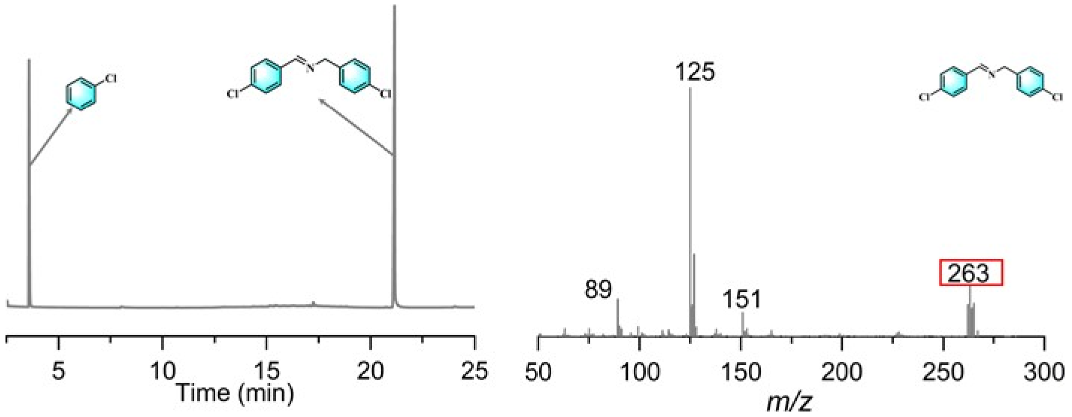

Figure S13. Gas chromatogram and mass spectrum authentication of N-(4-bromobenzyl)-1-(4-bromophenyl) methanimine obtained from the GC-MS analysis after 18 h reaction over the **Ti10** sample.

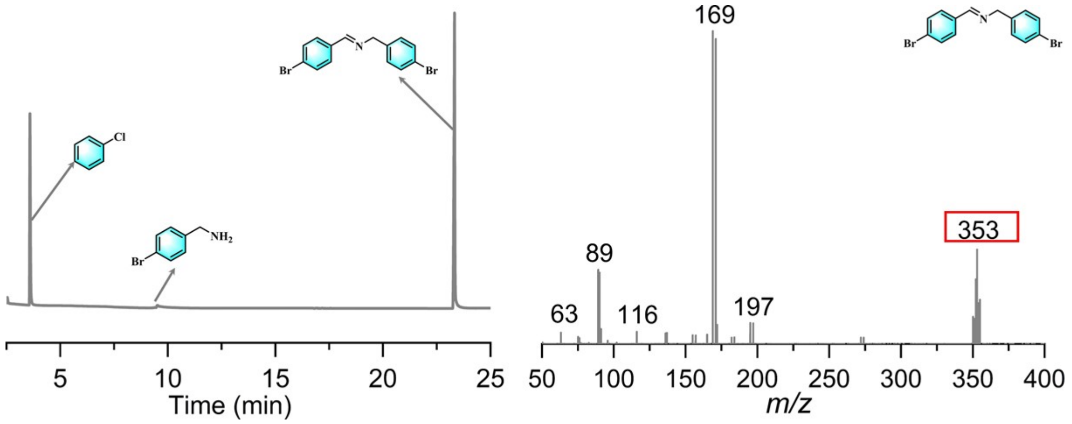

Figure S14. Gas chromatogram and mass spectrum authentication of N-(4-methylbenzyl)-1-(p-tolyl) methanimine obtained from the GC-MS analysis after 18 h reaction over the **Ti10** sample.

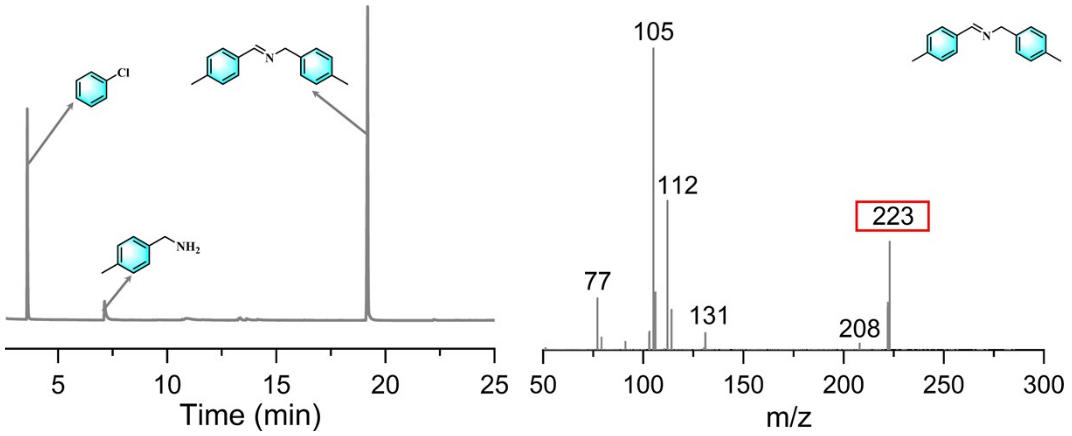

Figure S15. Gas chromatogram and mass spectrum authentication of N-(4-methoxybenzyl)-1-(4-methoxyphenyl) methanimine obtained from the GC-MS analysis after 18 h reaction over the **Ti10** sample.

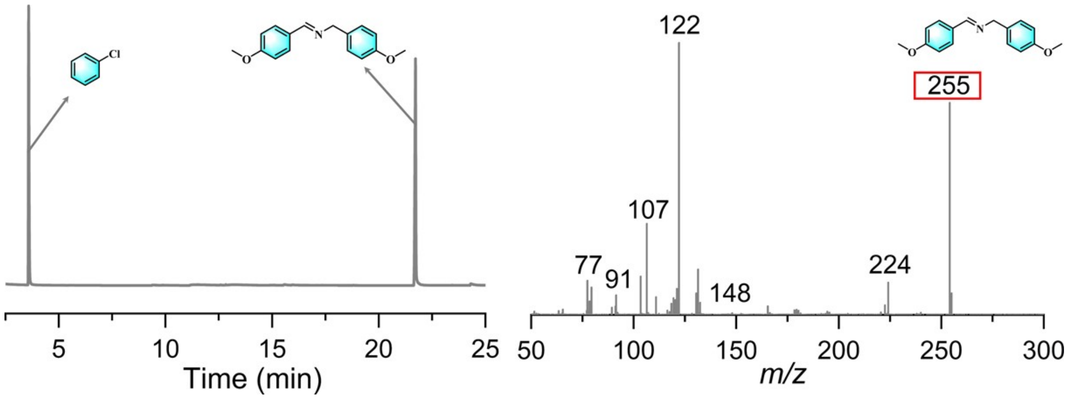

Figure S16. Gas chromatogram and mass spectrum authentication of N-(2-methoxybenzyl)-1-(2-methoxyphenyl) methanimine obtained from the GC-MS analysis after 18 h reaction over the **Ti10** sample.

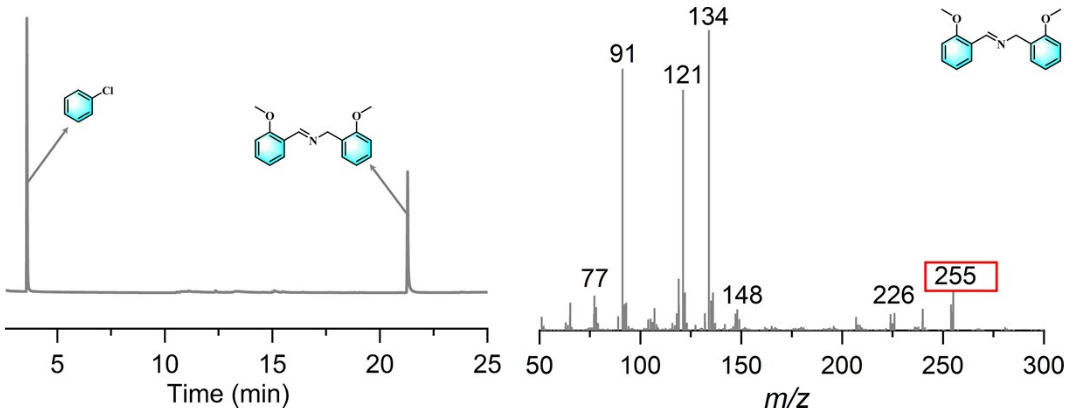

Figure S17. Gas chromatogram and mass spectrum authentication of N-(3-methoxybenzyl)-1-(3-methoxyphenyl) methanimine obtained from the GC-MS analysis after 18 h reaction over the **Ti10** sample.

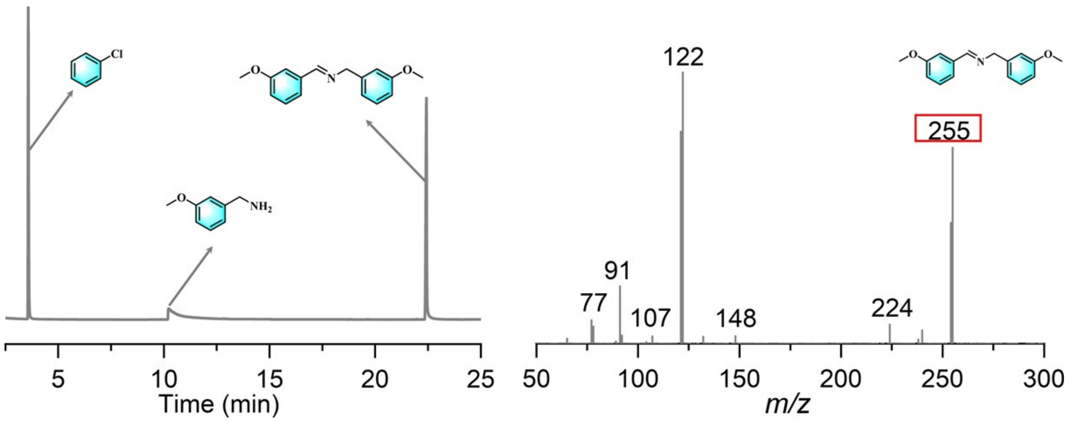

Figure S18. Gas chromatogram and mass spectrum authentication of N-(4-(tert-butyl) benzyl)-1-(4-(tert-butyl)phenyl) methanimine obtained from the GC-MS analysis after 18 h reaction over the **Ti10** sample.

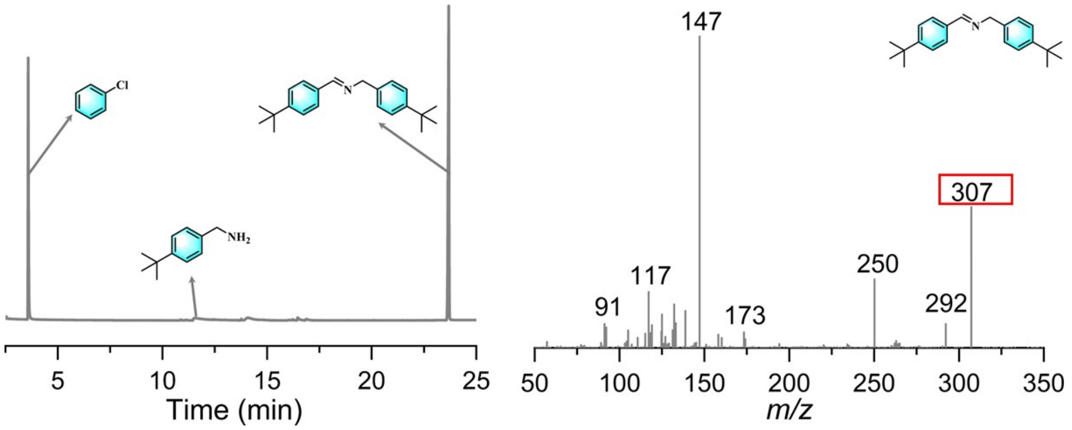

Figure S19. Gas chromatogram and mass spectrum authentication of N-(4-(trifluoromethyl) benzyl)-1-(4-(trifluoromethyl) phenyl) methanimine obtained from the GC-MS analysis after 18 h reaction over the **Ti10** sample.

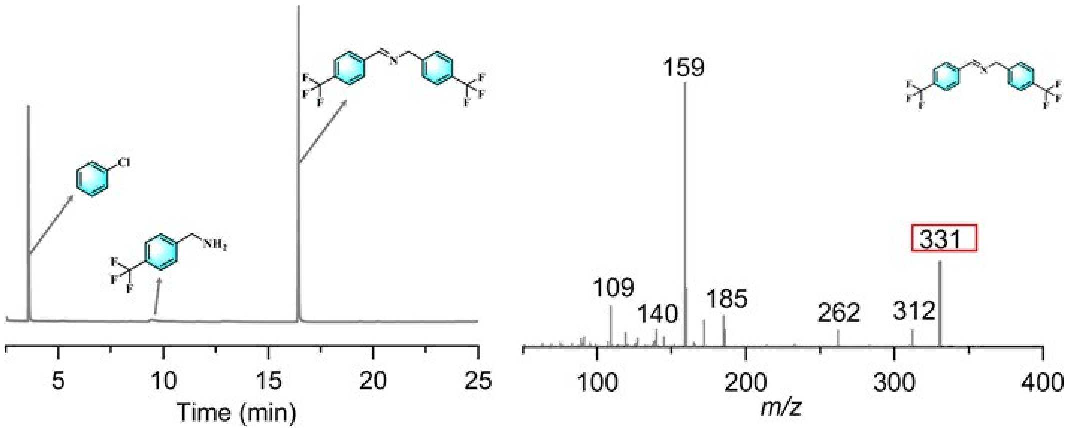

Figure S20. Gas chromatogram and mass spectrum authentication of (1-(thiophen-2-yl)-N-(thiophen-2-ylmethyl) methanimine obtained from the GC-MS analysis after 18h reaction over the **Ti10** sample.

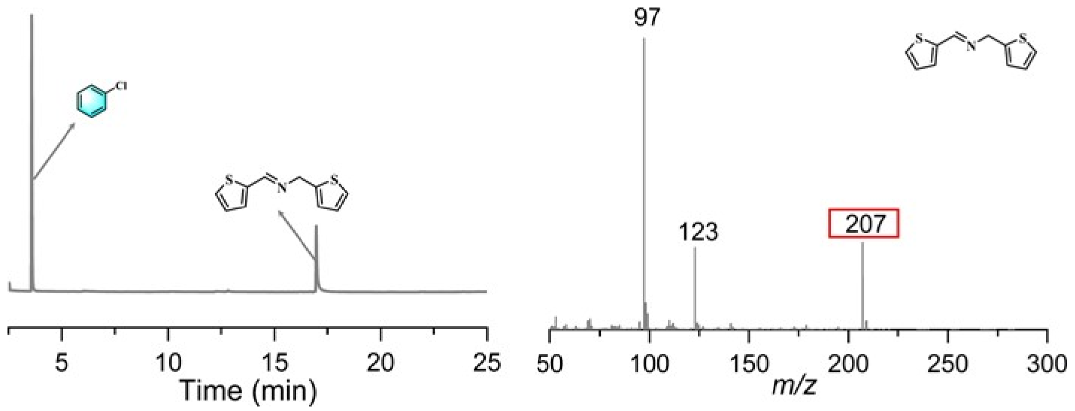

Reference

1 Q.-R. Ding, G.-L. Xu, L. Zhang, J. Zhang and Q.-R. Ding, Ligand-directed assembly engineering of trapezoidal {Ti_5} building blocks stabilized by dimethylglyoxime, *Dalton Trans.*, 2019, **48**, 9916-9919.

2 I. D. Brown, Recent Developments in the Methods and Applications of the Bond Valence Model, *Chem. Rev.*, 2009, **109**, 6858-6919.

3 W.-L. Sheng, F.-W. Huang, X.-Y. Dong, and X.-J. Lang, Solvent-controlled synthesis of Ti-based porphyrinic metal–organic frameworks for the selective photocatalytic oxidation of amines, *J. Colloid Interf. Sci.*, 2022, **628**, 784-793.

4 C.-Y. Xu, H. Liu, D.-D. Li, J.-H. Su and H.-L. Jiang, Direct evidence of charge separation in a metal–organic framework: efficient and selective photocatalytic oxidative coupling of amines via charge and energy transfer, *Chem. Sci.*, 2018, **9**, 3152-3158.

5 Z. Wang, X.-J. Lang, Visible light photocatalysis of dye-sensitized TiO_2: The selective aerobic oxidation of amines to imines, *Appl. Catalysis B: Environ.*, 2018, **224**, 404-409.

6 D.-R. Sun, L. Ye and Z.-H. Li, Visible-light-assisted aerobic photocatalytic oxidation of amines to imines over NH_2-MIL-125(Ti), *Appl. Catal., B*, 2015, **164**, 428-432.

7 W.-L. Sheng, J.-L. Shi, H.-M. Hao, X. Li and X.-J. Lang, Polyimide-TiO_2 hybrid photocatalysis: Visible light-promoted selective aerobic oxidation of amines, *Chem. Eng. J.*, 2020, **379**, 122399.

8 Y. Keum, B. Kim, A. Byun and J. Park, Synthesis and Photocatalytic Properties of Titanium-Porphyrinic Aerogels, *Angew. Chem., Int. Ed.*, 2020, **59**, 21591-21596.

9 Q. Niu, Q. Huang, T.-Y. Yu, J. Liu, J.-W. Shi, L.-Z. Dong, S.-L. Li, and Y.-Q. Lan. Achieving High Photo/Thermocatalytic Product Selectivity and Conversion via Thorium Clusters with Switchable Functional Ligands, *J. Am. Chem. Soc.*, 2022, **144**, 18586−18594.

10 E.-M. Han, W.-D. Yu, L.-J. Li, X.-Y. Yi, J. Yan and C. Chao, Accurate assembly of ferrocene-functionalized {$Ti_{22}Fc_4$} clusters with photocatalytic amine oxidation activity, *Chem. Commun.*, 2021, **57**, 2792-2795.

11 Y.-Q. Tian, Y.-S. Cui, W.-D. Yu, C.-Q. Xu, X.-Y. Yi, J. Yan, J. Li and C. Liu, An

ultrastable Ti-based metallocalixarene nanocage cluster with photocatalytic amine oxidation activity, *Chem. Commun.*, 2022, **58**, 6028–6031.
